# Study of the Evolution of the Plastic Zone and Residual Stress in a Notched T-6061 Aluminum Sample

**DOI:** 10.3390/ma15041546

**Published:** 2022-02-18

**Authors:** Luis Manuel Palacios-Pineda, José Emiliano Hernandez-Reséndiz, Oscar Martínez-Romero, Roque J. Hernandez Donado, Jesús Tenorio-Quevedo, Isaac H. Jiménez-Cedeño, Cecilio López-Vega, Daniel Olvera-Trejo, Alex Elías-Zúñiga

**Affiliations:** 1 Tecnológico Nacional de Mexico/Instituto Tecnológico de Pachuca, Pachuca 42080, Mexico; emi_jose.18@tec.mx; 2 Tecnologico de Monterrey, Department of Mechanical Engineering and Advanced Materials, School of Engineering and Sciences, Monterrey 64849, Mexico; oscar.martinez@tec.mx (O.M.-R.); isaac.jimenez@tec.mx (I.H.J.-C.); daniel.olvera.trejo@tec.mx (D.O.-T.); 3 Facultad de Ingeniería Mecánica, Universidad Autónoma del Caribe, Barranquilla 080020, Colombia; roque.hernandez@uac.edu.co; 4 Honeywell Aerospace, Vialidad Tabalaopa 8507, Parque Industrial Ávalos, Chihuahua 31074, Mexico; jesus.tenorioquevedo@honeywell.com (J.T.-Q.); cecilio.lopez@honeywell.com (C.L.-V.); 5 Vertiv, Ave. Luis Guadalupe Fernandez 3502, Parque Industrial FINSA, Santa Catarina 66380, Mexico

**Keywords:** plastic zone evolution, digital image correlation, residual stress

## Abstract

Using experimental measurements and numerical computations, this paper focuses on studying the evolution of the plastic zone and how the residual stresses change in a notched T-6061 aluminum sample. Before the crack initiation, digital image measurements were taken to visualize the evolution of the plastic zone. After the sample was fractured, the material microstructure and the residual stresses around the cracked zone were characterized through optical microscopy and X-ray diffractometry. This article describes in detail how the plastic zone evolved around the notch before the crack initiation and shows the close agreement between experimental and numerical data during the load increment. The surface residual stress values around the tip of the notched sample were also measured and computed to give a better understanding of the affected region during the fracture process.

## 1. Introduction

The reliable design of mechanical components is a great need for industry when security requirements are added, not only to safeguard infrastructure but also to avoid the loss of human lives. The fundamental requirement of any engineering system is that it does not fail in service. In this sense, digital image correlation has been used in recent years to monitor structural elements to help obtain more reliable mechanical components [1].

Although no statistics are available for catastrophic events, it is evident that the fracture problem is now more drastic than in previous centuries. Gordon established that the worst scenario in an engineering material is not a lack of strength or lack of rigidity, but a lack of resistance to crack propagation [2]. Therefore, there is a need to analyze engineering systems to predict the evolution of cracks in mechanical parts in order to maximize the component’s life and performance, and to provide the right time for replacement thereof before causing severe problems.

The parameter that characterizes stress in the area surrounding a crack tip is called the stress intensity factor (*K*) and depends on the geometry of the cracked part and the applied load [3]. All these factors have led to the performance of analyses to assess the behavior of the crack and predict the integrity of the structural component to prevent failure. Studies on the effect of intensity factor and critical stress fractures are reported in [4,5]. Since 2010, contactless measurement techniques based on digital image correlation have been used in the analysis of experimental mechanics for the aeronautical, automotive, and medical industries [6,7,8,9,10,11].

Different measurement methods have been used for studying the crack behavior, such as infrared techniques [12], X-ray tomography with digital volume correlation [13], and digital image correlation (DIC). Zhang and Liu used DIC to perform in situ optical microscopy fatigue testing to investigate the forward and reversed plastic zone size under cyclic loadings [14]. They observed that the crack closure significantly affected the reversed plastic zone size. Vasco-Olmo et al. presented a methodology for the experimental quantification of the crack tip plastic zone during fatigue crack growth using DIC [15]. Besel and Breitbarth studied the different types of plastic zones located at a crack tip under the cyclic loading of an aluminum alloy based on three-dimensional finite element simulations and mechanical testing. By digital image correlation and coupled FEM simulations, they analyzed the plastic zone at a crack tip of aluminum alloy AA2024-T3 [16,17]. Zhao and coworkers recently applied the DIC technique to obtain the continuous strain distribution at and in front of the crack tip as well as on the plastic zone ahead of the specimen crack tip [18].

Toribio and coauthors performed numerical analyses of plasticity-induced fatigue crack growth for high-strength steels and found that the plastic crack advancement was caused merely by plasticity reasons with no account for material decohesion or any rupture event at the crack tip [19]. Jia et al. studied two series of specimens, where they are respectively placed under pure shear, combined shear, and normal stress loading. They proposed a ductile fracture model with crack initiation, crack propagation rules, and deterioration of stress carrying capacity [20]. Yue and Wu evaluated the effects of various factors on the fracture parameters, crack initiation angles, and plastic zones of thin-walled cylindrical shells with cracks. They found that the stress in front of a crack tip did not increase after reaching the yield strength and entered the stage of plastic development [21].

The main objective of this work was to investigate the evolution of the plastic region generated around a specimen cracked zone using finite element simulations and experimental measurements via DIC. The specimen’s surface residual stresses were measured using an X-ray diffractometer and the collected data were compared with FEM numerical simulations to garner insight into the component structural changes that occur during the fracture process. It is important to point out that most of the wrought products produced in aerospace sector linear or rounded indications shall be permitted in any one-inch area and must not exceed certain length or diameter to fulfill the nonrotating components acceptance criteria. Therefore, we followed the ASTM-399 standard [22] to introduce a sample geometry with a linear indication that fulfills the criteria of a length-to-width ratio greater than three to one to determine the part residual stresses play via X-ray diffraction measurements.

## 2. Material Description, Experimental Procedure, and Numerical Computation

### 2.1. Material

**Figure 1:**
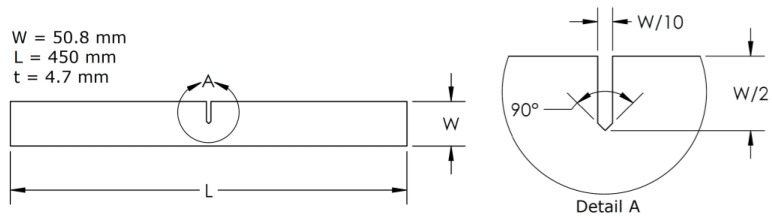
Tensile test specimens’ geometry in accordance with ASTM-399 [22].

We used a notched specimen made from aluminum alloy 6061 T5 obtained from a 50.8 mm wide and 4.7 mm thick flat bar, with shape and dimensions according to the ASTM-399 standard [22]; the sample’s geometry is shown in Figure 1. To obtain the notched specimen, plasma cutting was used to reduce material deformation and stress concentrations, and to prevent a change in the material properties due to cutting-induced temperature increment.

The various types of specimens that comply with ASTM standards can be categorized based on the following variables to assess their dependence on the fracture behavior: in-plane geometry, notch length, specimen size, specimen thickness, loading configuration, and notch orientation relative to material manufacture process configuration.

### 2.2. Tensile Test

**Figure 2:**
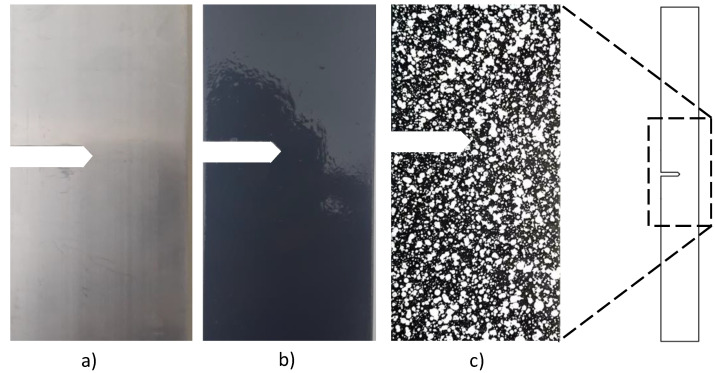
Surface specimen preparation for digital image correlation measurement: (**a**) clean material surface, (**b**) black painting, and (**c**) white speckled painting.

Once the specimens were prepared according to the ASTM standard, they were subjected to uniaxial tension test in a Shimadzu universal testing machine, model AG-IC 100 kN (Shimadzu, Kyoto, Japan). The crosshead speed was set to 5 mm/min with a frequency sample rate of 20 Hz. Digital image correlation (DIC) was used to measure the surface deformation and to obtain a complete strain field around the notched region, as shown in Figure 2.

We measured using DIC, with several images taken during experimental testing, and used correlation algorithms [5] to obtain the specimen’s strain and deformations. To adequately prepare the sample surface and get accurate measurements, the material surface around the notched region was painted to obtain a random pattern of black and white stains; the preparation steps are depicted in Figure 2. The random pattern allowed us to track the movement of pixels in the image for further analysis. Thanks to the speckle, the software could accurately identify and calculate the displacements of pixels, obtaining results with low error rates and high correlation coefficients [23].

**Figure 3:**
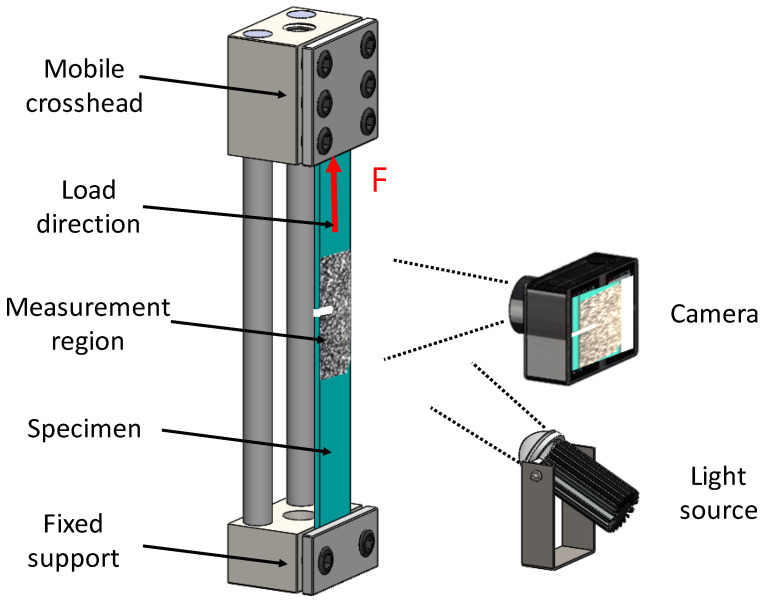
Experimental setup used in the strain measurements during the tensile test. It consisted of a video camera with a 5184 × 3456 pixel resolution with a frequency rate of 30 fps, and a light source.

Figure 3 illustrates the experimental setup used during the tensile test’s strain measurements. It consisted of the universal testing machine, a video camera with a 5184 × 3456 pixel resolution with a frequency rate of 30 frames per second, and a light source.

### 2.3. Digital Image Correlation

The image correlation process started with a grid containing control points. With these control points, a sub-image was defined as a reference image (undeformed specimen). The distorted image was analyzed as a sub-image with a shifted position to finally locate in sequence with the undeformed image and set the displacements by position vectors. The algorithm in Equation (1) gives results in displacement for each video frame through the tensile test:

(1) $$C\left(x,y,u,v\right)=\overset{n/2}{\underset{i,j=n/2}{\sum }}{\left(I\left(x+i,y+j\right)-I^{*}\left(x+u+i,y+v+j\right)\right)}^{2}$$

where x and y are the coordinates of the reference pixel, u and v are offset in the *x* and *y* directions, respectively, I is the image before the motion, and $I^{*}\;$is the image after the motion.

### 2.4. Residual Stress Measurement

**Figure 4:**
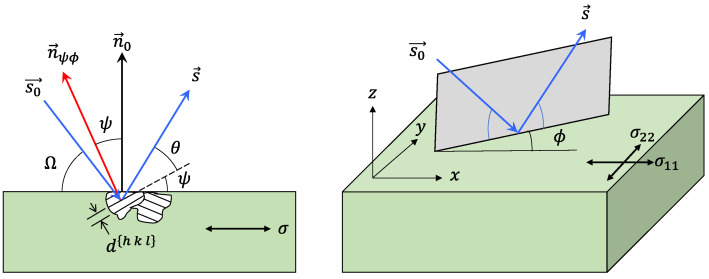
Netplanes of the lattice belong to the same {hkl} set. Strains are present in the crystal. X-ray diffraction angles: ψ, ϕ orientation angles of the netplane (to be measured), θ diffraction angle of the {hkl} plane (Bragg angle, to be measured), Ω incident angle, ${\vec{n}}_{\psi \phi }$ normal vector of the netplane, ${\vec{n}}_{o}$ normal vector of the surface, ${\vec{s}}_{o}$ incident (primary) X-ray beam, $\vec{s}$ diffracted X-ray beam.

The change in the material lattice parameter caused by sheet deformation is due to the change of position of a diffracted plane and is the measurement operating principle of XRD, see Figure 4. The apparatus irradiates X-rays over the surface to be measured and penetrates the sample, diffracting the incident X-rays. The deformed material changes its lattice parameter, following Bragg’s law. The residual stress measurements are on the surface as a result of the X-rays’ restricted energy. There is a plane stress condition on the sample surface and the stress tensor is described by the principal stresses $\sigma_{1}$ and $\sigma_{2}$. Consequently, the stress in a direction perpendicular to the surface has the value $\sigma_{3}=0$. It is essential to bear in mind that, due to Poisson’s ratio, there is a strain component $\varepsilon_{3}$ with a value different from zero in the direction perpendicular to the surface.

The following relationships were used to compute the residual stress from the X-ray measurements:

(2) $$\varepsilon_{\varphi \psi }^{\left\{hkl\right\}}=-\frac{1}{2}\cot\theta_{0}\left(2\theta_{\varphi \psi }-2\theta_{0}\right)$$

(3) $$\varepsilon_{\varphi \psi }^{\left\{hkl\right\}}=\frac{1}{2}s_{2}^{\left\{hkl\right\}}\left[\sigma_{11}\cos^{2}\varphi {\;\sin}^{2}\psi +\sigma_{22}\cos^{2}\phi {\;\sin}^{2}\psi +\sigma_{33}\cos^{2}\psi \right]\;+\frac{1}{2}s_{2}^{\left\{hkl\right\}}\left[\sigma_{12}\sin\left(2\phi \right){\;\sin}^{2}\psi +\sigma_{13}\cos\phi \sin\left(2\psi \right)+\sigma_{33}\cos^{2}\psi \right]+s_{1}^{\left\{hkl\right\}}\left[\sigma_{11}+\sigma_{22}+\sigma_{33}\right]$$

(4) $$\varepsilon_{\varphi \psi }^{\left\{hkl\right\}}=-{\left(\frac{\nu }{E}\right)}_{\left\{hkl\right\}}$$

(5) $$s_{2}^{\left\{hkl\right\}}={\left(\frac{1+\nu }{E}\right)}_{\left\{hkl\right\}}$$

where ψ represents the inclination angle of the line along which the lattice strain is computed; φ, $\sigma_{ii}$, and $\sigma_{ij}\;\left(i\neq j\right)$ represent the azimuth angle, the axial stress components, and the shear stress components, respectively; $\theta_{0}$ is the diffraction angle of the unstrained sample; and θ is the diffraction angle of the strained sample.

The spin of the finished part was not required during the X-ray measurements since we used the omega method. This method allowed us to analyze the complete part without the need of cutting it into sections. Therefore, the residual strain magnitudes could be measured with a high degree of accuracy by keeping the azimuth angle φ fixed, whereas the polar angle ψ changed. For X-ray diffraction measurements performed in a stress-free surface, the angle φ=0°, and thus the stress components $\sigma_{33}=0$, $\sigma_{12}=0$, and $\sigma_{23}=0$. Therefore, Equation (1) can be written as:

(6) $$\varepsilon_{\phi \psi }^{\left\{hkl\right\}}=\frac{1}{2}s_{2}^{\left\{hkl\right\}}\sigma_{11}\sin^{2}\psi +s_{1}^{\left\{hkl\right\}}\left[\sigma_{11}+\sigma_{22}\right].$$

Equation (6) can be written as follows since the material is isotropic:

(7) $$\varepsilon_{\varphi \psi }=\left(\frac{1+\nu }{2E}\right)\sigma_{11}\sin^{2}\psi +\left(\frac{\nu }{E}\right)\left[\sigma_{11}+\sigma_{22}\right].$$

The X-ray measurements of the lattice strain $\varepsilon_{\varphi \psi }$ were recorded in different directions of the angle ψ and plotted versus sin(2ψ). From the slope of the linear fit of Equation (7), the residual stress values could be calculated from the following expression:

(8) $$\sigma_{11}=\left(\frac{2E}{1+\nu }\right)\frac{\partial \varepsilon_{\varphi \psi }}{\partial \sin^{2}\psi }$$

as described by Grellner et al. [24], Hutchings et al. [25], and Jimenez et al. [26,27].

In this case, we required the analysis of a diffracted plane positioned above 120° in 2θ. We used a Panalytical Empyrean X-ray diffractometer (Malvern Panalytical, Malvern, UK) using α-Cu radiation, at 45 kV and 20 mA, with a *k-β* nickel filter to perform the experimental measurements. We scanned the material from 20° to 150° in 2θ to identify the peak value.

**Figure 5:**
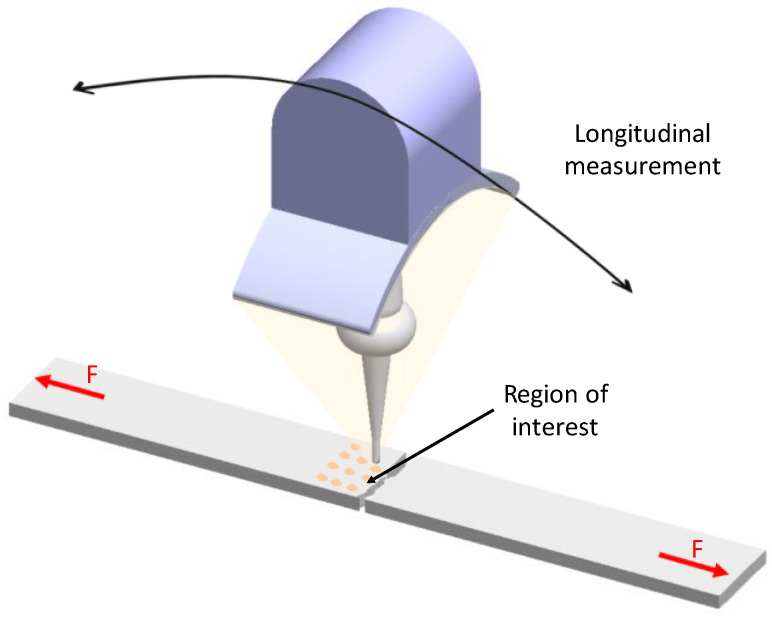
XRD measurement set up to measure residual stresses.

The sample setup in the diffractometer is illustrated in Figure 5. A total of nine locations were analyzed to obtain the residual stress by using the omega scanning mode.

We performed scans at different θ values, including the selected peak value of 137.5°, to determine the diffraction peak shift. This allowed us to find the corresponding shift value in the diffraction peak needed to compute the residual stresses. Because of the measurement method used, the X-ray beam optics were turned to avoid possible sample placing perturbations. The collected data from the scanning process were analyzed using the software Stress V1.

### 2.5. Finite Element Model

**Figure 6:**
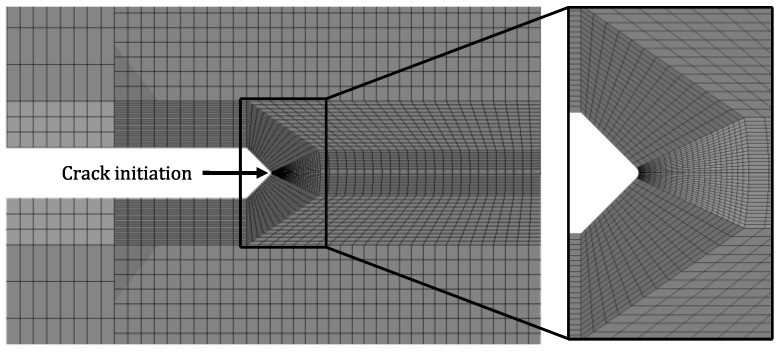
Finite element model used to compute the stress intensity factor and the plastic behavior during and after the test.

The ANSYS computer package was used to build the finite element model (FEM) around the notch to capture the evolution of the plastic zone and calculate the residual stresses. With the same geometry used in the experiments, the numerical model was developed with the mesh refinement needed to capture the notch’s stress gradient. Figure 6 illustrates the homogeneous mesh built around the notch. The simulations were performed with the multilinear isotropic hardening plasticity model since large strain values are expected without cycling loading. Since the model considers plastic behavior, it was possible to capture the plastic zone growth as the crack propagated.

## 3. Results

### 3.1. Experimental Tensile Tests

**Figure 7:**
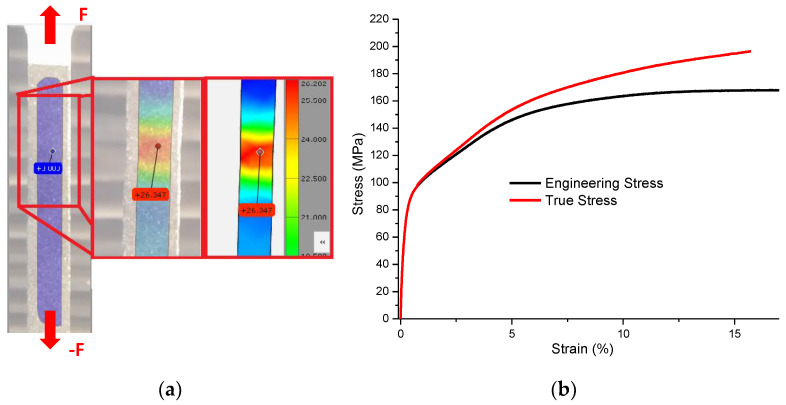
(**a**) Digital image correlation measurement during the tensile test. (**b**) Experimental stress–strain curve depicting the engineering and real stress. This plot shows the real strain captured from the digital image correlation (DIC).

Tensile tests were performed at constant jaw speed in the universal testing machine Shimadzu AG-IC 100 kN [28]. To measure the strain field, a set of 30 images per second were acquired by the digital image correlation tool (DIC) GOM Aramis Correlate Measurement System (GOM, Braunschweig, Germany). The graphical acquisition of the data from image correlation shows the evolution of the strain as the load increased. The colored scale shown in Figure 7a provides the distribution of the strain on the surface of the sample. The colored map illustrates how the sample central zone was highly strained in a uniform horizontal band, which indicates the appropriate sample geometry to avoid the strain concentration near the jaws, taking advantage of Saint-Venant’s principle. Figure 7b shows the engineering stress versus true strain curve obtained from the tensile test, as well as the true stress versus true strain curve to be used in the finite element nonlinear analysis. Table 1 summarizes the aluminum mechanical properties that were experimentally obtained from the three tested samples.

### 3.2. Notched Specimen Tensile Test

**Figure 8:**
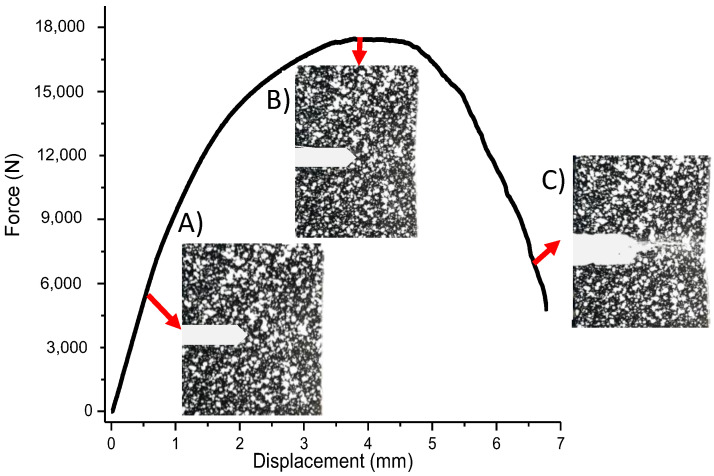
Force versus the crosshead displacement on the notched specimen and its correspondent crack opening.

Figure 8 shows the load applied to three single edge notched specimens whose experimental tests were performed with a feed rate of 5 mm/min and a machine with 20% sensitivity at break.

The result of the strain in the *y* direction shows an increase of up to 15% before the crack propagated as shown in Figure 8. This is because the specimen reached the last tensile strength found with specimens analyzed under the ASTM E8 standard. It is also argued that the mesh in the ARAMIS software breaks to reach the value of the ultimate tensile stress; this phenomenon occurred in all three tests on specimens of ASTM 399. This had a greater impact on the developed visual results in digital image correlation and finite element method.

### 3.3. Notched Specimen Plastic Region Evolution

**Figure 9:**
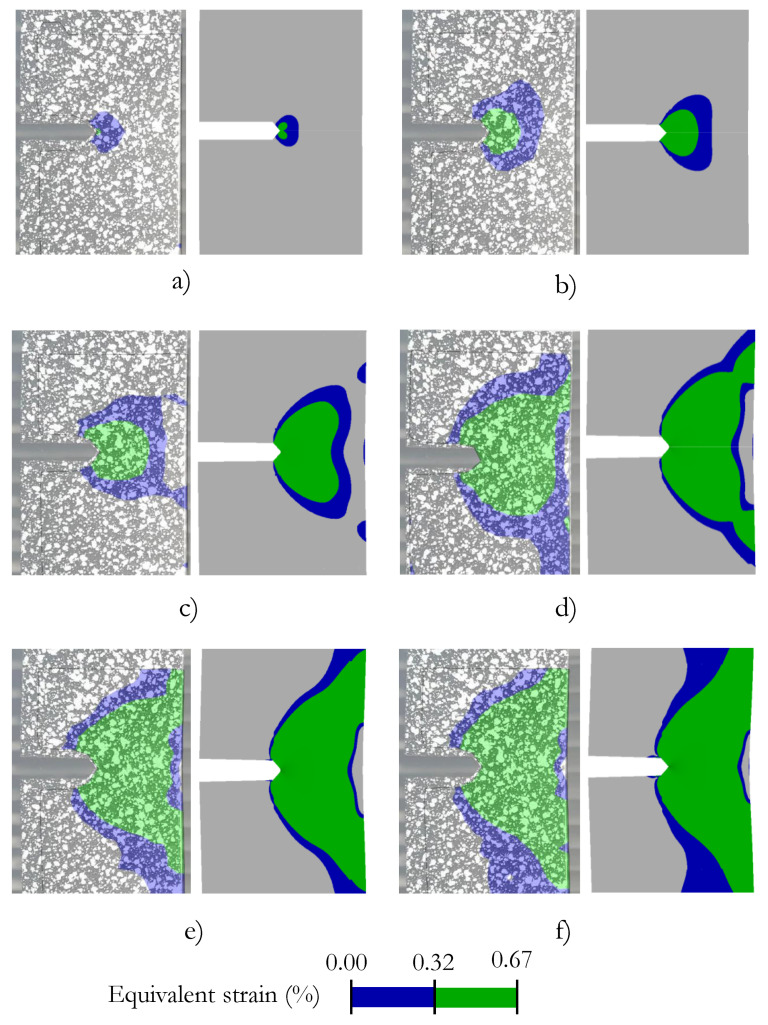
Evolution of the plastic region around the notched specimen. Comparison between the DIC experimental measurements and numerical predictions. Load of (**a**) 7.5 kN, (**b**) 11.2 kN, (**c**) 13.1 kN, (**d**) 15.3 kN, (**e**) 16.5 kN, and (**f**) 17.5 kN.

Here we provide a comparison of the plasticity region between digital image correlation measurements and numerical computation, using the finite element method software ANSYS, to assess the evolution of the sample plasticity before the unstable crack growth. Figure 9 shows the evolution of plastic deformation around the notch. The predicted plastic zone silhouette was in good agreement with the experimental measurements.

### 3.4. Determination of Residual Stresses

**Figure 10:**
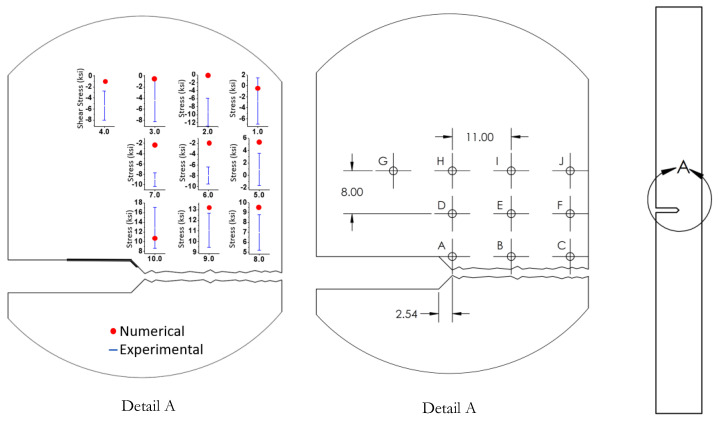
Comparison of the residual stress measurements with the numerical values in 9 points located in the vicinity of the initial crack tip as illustrated.

Residual stress measurements were carried out after the notched specimens were broken into two parts. Experimental measurements required the analysis of a diffracted plane positioned above 120° in 2θ. To identify the peak value, a scanning of the material from 20° to 150° in 2θ was performed. Following standard procedures, a peak value located at approximately 137.5° (2θ) was selected for the measurements. Figure 10 shows a comparison of the residual stress measurements with numerical values. The locations of the nine different zones where measurements were recorded are illustrated in Figure 10, in which it is evident that there was some variation between experimental tests and numerical predictions.

### 3.5. Fracture Surface

**Figure 11:**
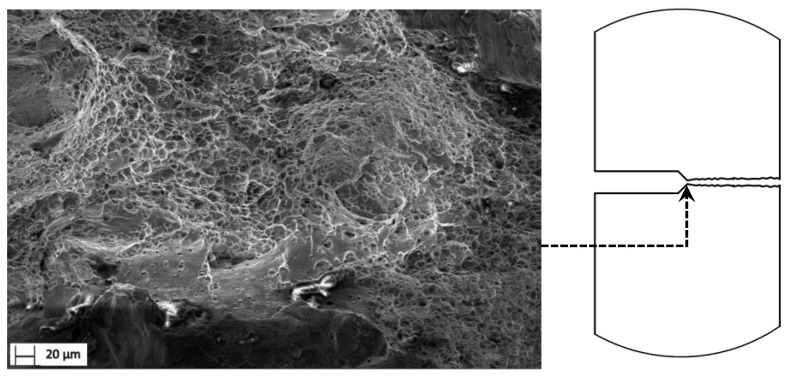
Ductile fracture morphology of specimen in the crack initiation region.

Tensile fracture surfaces are helpful in elucidating the effects of microstructure on the strength, ductility, and fracture properties of a material. Fractography of the tensile samples revealed that the macroscopic fracture morphology matched with that of a ductile fracture. Figure 11 shows SEM micrographs of the fracture surface. A ductile failure mode and the fracture surface was covered with two dimple categories: a low-density thick dimple and a higher-density dimple.

**Figure 12:**
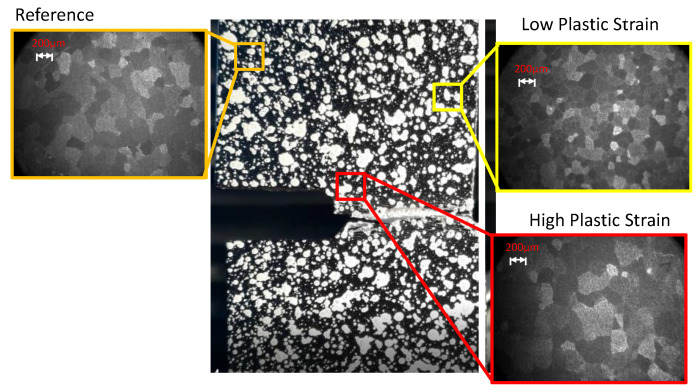
The microstructure at locations with low and high plastic strain.

Coarse constituent particles were observed in the bottom of the dimples, indicating the nucleation, growth, and coalescence of voids in relation to these particles. Voids are nucleated around the constituent particles or by cracking particles decohesion [29,30]. Even if particles were observed across the thick dimples, it cannot be excluded that the particles were present; in fact, the particles may have fallen during the fracture or may have been present on the surface opposite to the one in which the fracture’s origins initiated. Moreover, it is well-known that the plastic strain needed for the failure of a particle (or grain) increases with decreasing grain size. This could be explained by the smaller number of dislocations piling up at grain and phase boundaries. This results in lower shear stresses because of the length of the crack, which determines that the peak stress at the crack tip is restricted by the grain size. Thus, grain refinement increases cleavage fracture stress and promotes ductile fracture mechanisms at room temperature (Figure 12).

## 4. Conclusions

This paper elucidated the experimental and numerical procedures needed to measure and calculate the plastic region in notched aluminum specimens. Results confirmed that image correlation can be used as a practical alternative to measuring the evolution of the plastic zone around the crack tip. In summary, digital image correlation is a very practical tool to assess fracture and its behavior. This article described in detail how the plastic zone evolved around the notch before the crack initiation and showed the close agreement between experimental and numerical data during the load increment. The surface residual stress values around the tip of the notched sample were also measured and computed to give a better understanding of the affected region during the fracture process.

## Figures and Tables

**Table 1:** Mechanical properties of aluminum 6063 T6 obtained from tensile tests.

Specimen	Young’s Modulus (GPa)	Yield Stress (MPa)	Yield Strain (%)	Ultimate Tensile Stress (MPa)	Ultimate Tensile Strain (%)
1	69.07	97.76	0.703	168.60	15.01
2	69.02	99.66	0.607	168.93	14.62
3	69.36	100.12	0.690	168.66	15.03
Average	69.15	99.18	0.667	168.73	14.89

## Data Availability

The data presented in this study are available on request from the corresponding author.
